# A phase 2, randomized, double-blind safety and pharmacokinetic assessment of respiratory syncytial virus (RSV) prophylaxis with motavizumab and palivizumab administered in the same season

**DOI:** 10.1186/1471-2431-10-38

**Published:** 2010-06-03

**Authors:** Pilar Fernández, Adrian Trenholme, Katia Abarca, M Pamela Griffin, Micki Hultquist, Brian Harris, Genevieve A Losonsky

**Affiliations:** 1 Hospital Clínico Universidad de Chile, Santiago, Chile; 2 Department of Paediatrics, Kidz First Hospital, Auckland University, Auckland, New Zealand; 3 Hospital Clínico Pontificia Universidad Católica de Chile, Santiago, Chile; 4 MedImmune, Gaithersburg, MD 20878, USA

## Abstract

**Background:**

Respiratory syncytial virus (RSV) is an important pathogen causing annual epidemics of bronchiolitis and pneumonia among infants worldwide. High-risk infants currently receive RSV prophylaxis with palivizumab, a humanized RSV monoclonal antibody (MAb). In preclinical in vitro and in vivo (cotton-rat model) studies, motavizumab, a new RSV MAb, was shown to have greater anti-RSV activity than palivizumab. Motavizumab is currently under review for licensing approval. Since both MAbs may be available concurrently, this study evaluated their safety and tolerability when administered sequentially during the same RSV season.

**Methods:**

Between April 2006 and May 2006, 260 high-risk infants were randomly assigned 1:1:1 to receive monthly intramuscular injections: 2 doses of motavizumab followed by 3 doses of palivizumab (M/P); 2 doses of palivizumab followed by 3 doses of motavizumab (P/M); or 5 doses of motavizumab (control). Adverse events (AEs, serious AEs [SAEs]), development of antidrug antibody (ADA), and serum drug trough concentrations were assessed.

**Results:**

Most children received all 5 doses (246/260 [94.6%]) and completed the study (241/260 [92.7%]). While overall AE rates were similar (mostly level 1 or 2 in severity), SAEs and level 3 AEs occurred more frequently in the M/P group (SAEs: 22.9% M/P, 8.4% P/M, 11.8% motavizumab only; level 3 AEs: 15.7% M/P, 6.0% P/M, 6.5% motavizumab only). This trend in AE rates occurred before and after switching from motavizumab to palivizumab, suggesting a cause other than the combined regimen. Frequencies of AEs judged by the investigator to be related to study drug were similar among groups. Two deaths occurred on study (both in the M/P group, before palivizumab administration); neither was considered by the site investigator to be related to study drug. Mean serum drug trough concentrations were comparable among groups; ADA detection was infrequent (5.1% or less of any group).

**Conclusions:**

The conclusions drawn from this study are limited by the small sample size per group. However, within this small study, overall AE rates, serum drug trough concentrations, and development of ADA associated with administering motavizumab and palivizumab sequentially to high-risk children appear comparable to administering motavizumab alone during the same RSV season.

**Trial Registration:**

clinicaltrials.gov NCT00316264

## Background

Respiratory syncytial virus (RSV) is an important respiratory pathogen that produces annual epidemics of bronchiolitis and pneumonia in young children worldwide [1-3]. The greatest morbidity and mortality occur among children at high risk for severe RSV disease, including premature infants, infants with chronic lung disease (CLD), and infants with complicated congenital heart disease [4-6].

These high-risk infants currently receive prophylaxis for RSV with palivizumab (MedImmune, Gaithersburg, MD, USA), which is recommended and indicated for the prevention of severe RSV disease in high-risk children [7,8]. Palivizumab is a humanized monoclonal antibody that recognizes a highly conserved neutralizing epitope in the A antigenic site of the F glycoprotein of RSV [9]. Monthly palivizumab administration has been shown to reduce RSV hospitalizations by approximately 50% compared with placebo in high-risk children with prematurity, CLD of prematurity, and congenital heart disease [10-12]. In addition, in the IMpact study, preterm infants without CLD who received prophylaxis with palivizumab had an even greater reduction in RSV-related hospitalizations that approached 80% [12].

Motavizumab (MEDI-524, MedImmune) is an investigational monoclonal antibody developed by affinity maturation of palivizumab. Compared with palivizumab, motavizumab has an approximately 75-fold greater affinity for the RSV F protein [13,14], is approximately 20-fold more active in microneutralization studies, and, in the cotton rat model, reduces nasal and lung RSV titers 25- and 100-fold, respectively [14,15]. In comparison with controls, in a mouse model of RSV, motavizumab was also found to be associated with significant reductions in RSV replication and concentrations of cytokines (interleukin-1 alpha, interleukin-12p70 and tumor necrosis factor alpha, interferon gamma) that are probably related to improvements observed in clinical markers of disease severity [16].

Early pediatric clinical studies of motavizumab in which children received monthly intramuscular dosing demonstrated no dose-limiting toxicities, and serum pharmacokinetics were found to be consistent with published data for palivizumab [17,18]. In addition, in a proof-of-concept phase 1 study, children hospitalized with RSV illness were given a single dose of intravenous motavizumab or placebo [18]. In that study, motavizumab significantly reduced viral load and culturable RSV was eliminated 1 day post-treatment in a greater proportion of treated children compared to those who received placebo. Significant anti-RSV effects were not seen in the upper respiratory tracts of infants in a similar study conducted with palivizumab [19]. Recently, a large clinical study of over 6600 infants[20] to evaluate monthly immunoprophylaxis during the RSV season with motavizumab compared to palivizumab was completed. In this study of premature infants with and without CLD, motavizumab was shown to be noninferior to palivizumab with a 26% relative reduction in the primary endpoint of RSV hospitalizations (1.4% vs 1.9%, respectively; relative risk, 0.740; 95% confidence interval: 0.503, 1.083) [20]. In addition, in the outpatient setting, a secondary endpoint, motavizumab was superior to palivizumab, effecting a 50% relative reduction of RSV-specific, medically attended, lower respiratory tract infections (2.0% vs 3.9%, respectively; *P *= 0.005) [20].

Motavizumab is currently under review by the US Food and Drug Administration for the prevention of serious lower respiratory tract disease caused by RSV in children at high risk of RSV disease. Because it is likely that initially both products will be available concurrently for commercial use, there could be times when a child receives both agents sequentially within the same RSV season. Therefore, the primary objective of this study was to provide initial data on the safety of motavizumab and palivizumab when administered sequentially to high-risk, preterm infants during the same RSV season. Additional objectives of the study were to determine serum trough concentrations of motavizumab and palivizumab when administered during the same RSV season and to evaluate the development of antimotavizumab and antipalivizumab antidrug antibodies (ADA).

## Methods

### Study Design

This was a phase 2, randomized, double-blind study in which motavizumab and palivizumab were administered during the same RSV season to premature infants with and without CLD. The study was conducted between April 2006 and February 2007. During the 2006 RSV season, eligible infants were enrolled at 18 sites in the southern hemisphere, which included Chile (7 sites), New Zealand (5 sites), and Australia (6 sites). Eligible children were randomly assigned (1:1:1, stratified by site) to one of 3 treatment groups using an automated randomization system: 2 doses of motavizumab followed by 3 doses of palivizumab (M/P); 2 doses of palivizumab followed by 3 doses of motavizumab (P/M); or 5 doses of motavizumab only (control). Motavizumab and/or palivizumab were administered at 15 mg/kg by intramuscular injection every 30 days, for a total of 5 planned doses. These are the prescribed dose and regimen as currently used for palivizumab. Doses were administered on study days 0, 30, 60, 90, and 120. Drug administration of dose 2 (day 30) was allowed between day 25 and day 30. For all other doses, a prespecified ± 5-day window was allowed.

The study was conducted in accordance with the Declaration of Helsinki and in compliance with the ethical principles of the International Conference on Harmonization Guidelines for Good Clinical Practice. The study was approved by the institutional review board or independent ethics committee of each participating center. Parents or legal guardians provided written informed consent for each subject before enrollment.

### Study Endpoints

The primary endpoints were safety and tolerability of motavizumab and palivizumab administered sequentially during the same RSV season, as assessed by summarizing adverse events (AEs), serious AEs (SAEs), and laboratory evaluations. Secondary endpoints were ADA and serum trough concentrations of motavizumab and palivizumab. AEs were defined as any unfavorable and unintended sign (including an abnormal laboratory finding), symptom, or disease that occurred while the child was enrolled in the study from the time of randomization through study day 150, whether or not the event was considered to be related to the study treatment. SAEs were defined as AEs that resulted in death, risk of life, hospitalization or prolongation of existing hospitalization, persistent or significant disability or incapacity, or that required medical or surgical intervention to prevent any of these outcomes. AEs and SAEs were summarized by system organ class and preferred terms using the Medical Dictionary for Regulatory Activities (MedDRA), by severity (levels 1-4, which corresponded to mild, requiring no treatment; moderate, possibly requiring symptomatic therapy; severe, generally requiring a more immediate medical evaluation or treatment; and life-threatening, requiring immediate medical attention to support vital functions, respectively), and by relationship to study treatment (none, remote, possible, probable, definite). AEs were assessed by the investigator for severity, relationship to the study treatment, and whether the event met criteria as an SAE (ie, not all level 4 AEs, such as laboratory abnormalities, met criteria for SAE). All summaries of AEs include both nonserious AEs and SAEs.

### Study Inclusion and Exclusion Criteria

Preterm children were eligible to participate if the gestational age was ≤35 weeks *and *the chronologic age was ≤6 months at the time of entry into the study, or if they were ≤24 months of age at the time of entry into the study and had a diagnosis of CLD of prematurity requiring medical management within 6 months before randomization. Eligible children had to be in good health at the time of study entry. They could not be hospitalized (unless discharge was expected within 10 days); be receiving chronic oxygen therapy or any ventilatory support; have significant congenital heart disease; have evidence of infection with hepatitis A, B, or C virus or HIV; have any acute illness, including acute RSV infection; known renal impairment, hepatic dysfunction, chronic seizure disorder, or immunodeficiency; have suspected serious allergic or immune-mediated events in association with prior receipt of immunoglobulins, blood products, or other foreign proteins; have received within the past 120 days or currently be receiving immunoglobulin products, palivizumab, or any investigational agent.

### Study Assessments

Subjects were evaluated before each injection of study drug, at study day 150, which corresponded to 30 days after the fifth and final planned dose of study drug, and at the final follow-up visit between study days 270 and 300, which corresponded to 120 to 150 days after the fifth dose of study drug. Subjects were monitored for AEs and SAEs from the time of the first study drug administration through study day 150. Blood was collected to assess serum drug trough concentrations and development of ADA on day 0 (pre-drug baseline), day 60 (before dose 3), and day 150. Final assessments of antimotavizumab and antipalivizumab antibody and serum trough concentrations of motavizumab and palivizumab were performed between study days 270 and 300.

Enzyme linked immunosorbent assays to quantify ADAs and trough serum concentrations of motavizumab and palivizumab were developed by MedImmune and validated and performed by PPD Development, LP (Richmond, VA). These methods have been described previously [9,17]. No data on the prevention of RSV disease were collected.

### Study Products

Study drugs were provided in sterile, preservative-free vials containing 100 mg motavizumab in 1 mL 25 mM histidine-HCL, pH 6.0, or 100 mg palivizumab in 1 mL 25 mM histidine, 1.6 mM glycine, pH 6.0. Both study products were stored at 2° to 8°C. Motavizumab was administered at the same prescribed dose and regimen as is currently used for palivizumab (15 mg/kg administered by intramuscular injection at monthly intervals for 5 months).

### Statistical Methods and Analysis Populations

As no hypothesis testing was planned to compare treatments, no formal sample size calculations were performed. However, AE rates were compared between treatment groups using a 2-sided Fisher's exact test to note significant differences. Statistically significant differences were reported for *P *values < 0.05. Given the number of subjects enrolled and included in the safety analyses, the minimum statistical difference that could have been detected with 80% power between the control arm (N = 93) and either mixed-dose arm (N = 83) was 10 percentage points for event rates of 1.0% and 22 percentage points for event rates of 50%. For example, if one treatment group had an event rate of 1%, an event rate of at least 11% would have been necessary in the comparison treatment group to allow at least an 80% chance of detecting a statistically significant difference between the two treatment groups. Similarly, if one treatment group had an event rate of 50%, an event rate of at least 72% would have been necessary in the comparison treatment group. Due to the number of tests that were performed, the *P *values should be interpreted with caution. No adjustments for multiple comparisons were made.

Statistical analyses were performed using SAS/STAT^® ^Version 8.2. Categorical data are summarized by the number and percent of subjects in each category. Continuous variables are summarized by descriptive statistics including mean, standard deviation, minimum, and maximum. Study day 0 was defined as the day of randomization.

The intent-to-treat population included all randomized subjects. The safety population included all randomized subjects who received study drug and had any safety follow-up. The serum drug trough concentration and ADA population included all subjects in the safety population who did not receive commercial palivizumab during the 120 days before study day 0. Subjects were excluded from any individual time-point summary of serum drug trough concentration and ADA if they did not receive the correct number of study treatment doses before that time point.

## Results

### Study Population

A total of 260 subjects were enrolled into the 3 treatment groups during April and May 2006: M/P, n = 83; P/M, n = 84; and motavizumab only (control), n = 93; 259 subjects received at least one dose of study drug. Demographic characteristics at study entry are presented in Table 1. Overall, the treatment groups were balanced with respect to chronologic age, weight, and gestational age. There were 2 minor imbalances noted that were not felt to affect the outcome of the study: 1) the baseline incidence of CLD was slightly lower in the M/P treatment group (13.3%) than in the P/M and motavizumab-only (control) treatment groups (16.7% and 17.2%, respectively); and 2) there were slightly more male subjects enrolled in the M/P treatment group (61% in M/P compared with 51% and 52% in the P/M and the motavizumab-only [control] groups, respectively).

**Table 1:** Demographic characteristics at study entry

Characteristic	Mixed Motavizumab/Palivizumab (n = 83)	Mixed Palivizumab/Motavizumab (n = 84)	Motavizumab Only (n = 93)	Total (N = 260)
Age, mo				
Mean (SD)	3.7 (3.1)	3.4 (2.3)	3.9 (2.4)	3.7 (2.6)
Gestational age at birth, wk				
Mean (SD)	31.0 (2.6)	31.3 (2.9)	30.9 (2.7)	31.1 (2.7)
Sex, n (%)				
Male	51 (61.4)	43 (51.2)	48 (51.6)	142 (54.6)
Race/ethnicity, n (%)				
White/Non-Hispanic	23 (27.7)	20 (23.8)	28 (30.1)	71 (27.3)
Hispanic	53 (63.9)	56 (66.7)	57 (61.3)	166 (63.8)
Other	7 (8.4)	8 (9.5)	8 (8.6)	23 (8.8)
Weight, kg				
Mean (SD)	4.6 (2.0)	4.4 (1.8)^a^	4.7 (1.7)	4.6 (1.8)
CLD present, n (%)	11 (13.3)	14 (16.7)	16 (17.2)	41 (15.8)

^a^n = 83 (weight was not recorded for one subject).

### Subject Compliance, Disposition, and Discontinuations

**Figure 1:**
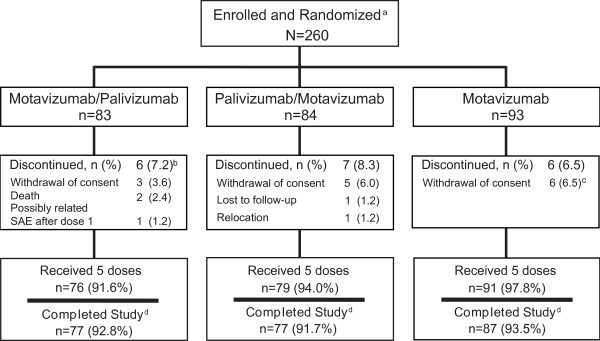
**Subject disposition through the end of the study period**. ^a^Intent-to-treat population. ^b^Includes 1 subject with an SAE of visual disturbance who discontinued drug after 1 dose and did not complete the study; 2 additional subjects discontinued drug after receiving < 5 doses of study drug (1 with erythema multiforme after 2 doses and 1 with staphylococcal scalded skin syndrome after 3 doses, both in the M/P group), but since they were followed through the end of the study period are classified as having completed the study, thus they are not included in this total. ^c^Includes 1 subject for whom consent was withdrawn on day 13 and who died of a bowel obstruction on day 153. ^d^Completed the study through study days 270-300 (120-150 days after the final dose).

Subjects who remained in the study for the final follow-up visit between study days 270 and 300 were deemed to have completed the study. The majority (246/260, 94.6%) of subjects received all 5 doses of study drug, and a total of 241/260 (92.7%) subjects completed the study (Figure 1). Two children who had study drug discontinued due to SAEs continued with follow-up until the completion of the study; both were in the M/P group (one developed erythema multiforme after 2 doses; another developed staphylococcal scalded skin syndrome after 3 doses). Another child who discontinued study drug after an SAE of visual disturbance (also in the M/P group) after receiving 1 dose of study drug did not continue in the study. All 3 of these children were included in the safety population. One subject assigned to the P/M treatment group did not receive study drug and was therefore not included in the safety, ADA, or serum drug trough concentration analyses.

### Safety: Adverse Events

A comparative overview of AEs for the 3 groups overall and by severity is presented in Table 2. Overall, 89% to 93% of subjects in all 3 groups experienced at least 1 AE. In general, AE rates were similar among the treatment groups, with the exception of level 3 events reported as the highest severity and SAEs, for which the M/P treatment group had more subjects with these events than the other 2 groups. In the M/P group, 15.7% reported at least one level 3 AE, compared with 6.0% in the P/M group and 6.5% in the motavizumab-only (control) group. The incidence of level 3 and level 4 AEs in the M/P group was significantly higher compared with the P/M group (19.3% vs. 6.0%, respectively; *P *< 0.05) but did not differ significantly from the rate in the motavizumab-only (control) group (10.8%). More than half of level 3 or level 4 events in any treatment group (15/17, 5/7, and 6/10 events in the M/P, P/M, and motavizumab-only [control] groups, respectively) were classified in the MedDRA "infections and infestations" system organ class. A total of 24 SAEs occurred in 19 children in the M/P group, 9 SAEs occurred in 7 children in the P/M group, and 12 SAEs occurred in 11 children in the motavizumab-only (control) group (Table 2).

**Table 2:** Comparison of adverse events overall and by severity

	Mixed Motavizumab/Palivizumab (n = 83)	Mixed Palivizumab/Motavizumab (n = 83)	Motavizumab Only (n = 93)
Total number of AEs	405	408	459
Number (%) of children reporting:			
≥1 AE	77 (92.8)	75 (90.4)	83 (89.2)
≥1 Level 1 AE, as the highest severity	17 (20.5)	26 (31.3)	22 (23.7)
≥1 Level 2 AE, as the highest severity	44 (53.0)	44 (53)	51 (54.8)
≥1 Level 3 AE, as the highest severity	13 (15.7)	5 (6.0)	6 (6.5)
≥1 Level 4 AE, as the highest severity	3 (3.6)	0 (0.0)	4 (4.3)
≥1 Related AE	14 (16.9)	16 (19.3)	21 (22.6)
≥1 SAE	19 (22.9)^a^	7 (8.4)^a^	11 (11.8)
≥1 Level 1 SAE, as the highest severity	1 (1.2)	0 (0.0)	0 (0.0)
≥1 Level 2 SAE, as the highest severity	4 (4.8)	4 (4.8)	4 (4.3)
≥1 Level 3 SAE, as the highest severity	11 (13.3)^a^	3 (3.6)^a^	4 (4.3)
≥1 Level 4 SAE, as the highest severity	3 (3.6)	0 (0.0)	3 (3.2)
≥1 Related SAE	2 (2.4)	0 (0.0)	1 (1.1)
≥1 AE resulting in discontinuation of study drug	3 (3.6)	0 (0.0)	0 (0.0)
≥1 Related AE resulting in discontinuation of study drug	2 (2.4)	0 (0.0)	0 (0.0)
Death	2 (2.4)	0 (0.0)	1 (1.1)^b^

^a^*P *< 0.05, mixed motavizumab/palivizumab vs. mixed palivizumab/motavizumab (2-sided Fisher's exact test; exploratory analysis only).

^b^This patient withdrew consent on study day 13 and died on study day 153.

Overall, the types and numbers of specific AEs were similar among the 3 treatment groups; those occurring in at least 5% of subjects in any group are summarized in Table 3. The most frequent AEs were infectious in nature and were expected for this population of high-risk children. The most commonly reported AEs included nasopharyngitis, upper respiratory infection, wheezing, conjunctivitis, teething, rhinitis, diarrhea, and bronchitis. Most AEs in any treatment group were level 1 or 2 in severity (M/P, 388/405 [95.8%]; P/M, 401/408 [98.3%]; and motavizumab only [control], 449/459 [97.8%]).

**Table 3:** Adverse events occurring at ≥5% of any treatment group at any severity level

	Mixed Motavizumab/Palivizumab (n = 83)	Mixed Palivizumab/Motavizumab (n = 83)	Motavizumab Only (n = 93)
**Total number of AEs**	405	408	459
**Number (%) of subjects reporting **≥**1 AE**	77 (92.8)	75 (90.4)	83 (89.2)

**Number (%) of subjects reporting AE by body system**^**a**^			
**Blood and lymphatic system disorders**			
Anaemia	2 (2.4)	6 (7.2)	3 (3.2)
**Eye disorders**			
Conjunctivitis	6 (7.2)	11 (13.3)	16 (17.2)
**Gastrointestinal disorders**			
Constipation	6 (7.2)	6 (7.2)	7 (7.5)
Diarrhea	12 (14.5)	13 (15.7)	11 (11.8)
Gastroesophageal reflux disease	5 (6.0)	7 (8.4)	7 (7.5)
Inguinal hernia	0 (0.0)	4 (4.8)	5 (5.4)
Teething	10 (12.0)	9 (10.8)	16 (17.2)
**General disorders and administration-site conditions**			
Injection site erythema	3 (3.6)	9 (10.8)	8 (8.6)
Irritability	10 (12.0)	11 (13.3)	11 (11.8)
Pyrexia	6 (7.2)	8 (9.6)	11 (11.8)
**Infections and infestations**			
Bronchiolitis	11 (13.3)	7 (8.4)	5 (5.4)
Bronchitis	12 (14.5)	13 (15.7)	13 (14.0)
Bronchitis acute	3 (3.6)	7 (8.4)	6 (6.5)
Gastroenteritis	8 (9.6)	4 (4.8)	5 (5.4)
Lower respiratory tract infection	7 (8.4)	7 (8.4)	6 (6.5)
Nasopharyngitis	25 (30.1)	26 (31.3)	23 (24.7)
Oral candidiasis	2 (2.4)	5 (6.0)	2 (2.2)
Otitis media acute	2 (2.4)	0 (0.0)	5 (5.4)
Pharyngitis	7 (8.4)	4 (4.8)	10 (10.8)
Rhinitis	10 (12.0)	14 (16.9)	8 (8.6)
Upper respiratory tract infection	17 (20.5)	16 (19.3)	18 (19.4)
**Laboratory investigation abnormalities**			
Alanine aminotransferase increased	1 (1.2)	1 (1.2)	5 (5.4)
**Nervous system disorders**			
Hypertonia	5 (6.0)	1 (1.2)	0 (0.0)
**Respiratory, thoracic, and mediastinal disorders**			
Cough	6 (7.2)	4 (4.8)	5 (5.4)
Nasal congestion	7 (8.4)	1 (1.2)	3 (3.2)
Rhinorrhoea	3 (3.6)	5 (6.0)	1 (1.1)
Wheezing	15 (18.1)	8 (9.6)	10 (10.8)
**Skin and subcutaneous disorders**			
Dermatitis diaper	5 (6.0)	10 (12.0)	10 (10.8)
Eczema	1 (1.2)	5 (6.0)	4 (4.3)
Rash	3 (3.6)	5 (6.0)	4 (4.3)
Seborrhoeic dermatitis	4 (4.8)	1 (1.2)	5 (5.4)

^a^MedDRA (version 9.1) system organ classes and preferred terms are shown.

Due to the higher frequencies of level 3 AEs (as the highest severity) and SAEs in the M/P treatment group, a post hoc analysis was performed to determine the incidence of AEs before and after receipt of dose 3 (when the switch to the second study drug occurred). It is important to note that the reporting period for this analysis encompassed 60 days before dose 3 and 90 days after dose 3. An overview of safety data before and after dose 3 is presented in Table 4. The increases in level 3 or 4 AEs and SAEs were consistently observed both before and after dose 3 in the M/P group. After 2 doses of study drug (ie, prior to dose 3), all groups had comparable rates of AEs and AEs deemed by the site investigators to be related to study treatment. Before dose 3, the rates of level 3 AEs as the highest severity, level 4 AEs, SAEs, SAEs judged by the investigator to be related to study drug, and AEs resulting in discontinuation of study drug were all higher among children in the M/P group than in the motavizumab-only (control) treatment group. Some of these differences persisted after receipt of dose 3 of study drug, with the M/P group exhibiting higher rates of level 3 AEs (as the highest severity) and SAEs compared with either the motavizumab-only (control) or the P/M treatment group. The number of children who experienced SAEs before and after dose 3 was similar for both dosing periods in each group. In the M/P group, 14 of the 24 SAEs were level 3 or level 4 in severity; similarly, 14 of the 17 level 3 or level 4 AEs were considered SAEs. In the P/M group, 4 of the 9 SAEs were level 3 in severity; likewise, 4 of the 7 level 3 AEs were considered SAEs. No level 4 AEs were reported in this group. In the motavizumab-only (control) group, 7 of the 12 SAEs were level 3 or level 4 in severity; similarly, 7 of the 10 level 3 or level 4 AEs were considered SAEs. At least one-half of SAEs in any treatment group (19/24 in the M/P group, 7/9 in the P/M group, 6/12 in the motavizumab-only [control] group) were infection-related. In the M/P group, a total of 12 of 24 SAEs occurred after receipt of dose 3 (ie, when subjects had received both study drugs). All 12 were infectious in nature, and 11 of the 12 were respiratory infections. Ten of the 11 respiratory infections had local viral testing performed, with only 2 tests positive for *Rsv*.

**Table 4:** Overview of safety data before and after dose 3

	Before Dose 3			After Dose 3		
		
	Mixed Motavizumab/Palivizumab (n = 83)	Mixed Palivizumab/Motavizumab (n = 83)	Motavizumab Only (n = 93)	**Mixed Motavizumab/Palivizumab (n = 78**^**a**^**)**	**Mixed Palivizumab/Motavizumab (n = 82**^**a**^**)**	**Motavizumab Only (n = 92**^**a**^**)**
AEs, n	175	186	204	230	222	255
Subjects reporting, n (%)						
≥1 AE	66 (79.5)	64 (77.1)	69 (74.2)	70 (89.7)	68 (82.9)	71 (77.2)
≥1 Level 3 AE as highest severity	3 (3.6)	3 (3.6)	1 (1.1)	10 (12.8)	3 (3.7)	5 (5.4)
≥1 Level 4 AE	3 (3.6)^b^	0 (0.0)	1 (1.1)	0 (0.0)	0 (0.0)	3 (3.3)^b^
≥1 Related AE	13 (15.7)	9 (10.8)	13 (14.0)	6 (7.7)	11 (13.4)	15 (16.3)
≥1 SAE	10 (12.0)	4 (4.8)	6 (6.5)	12 (15.4)	5 (6.1)	6 (6.5)
≥1 Related SAE	2 (2.4)	0 (0.0)	0 (0.0)	0 (0.0)	0 (0.0)	1 (1.1)
≥1 AE resulting in discontinuation of study drug	2 (2.4)^c^	0 (0.0)	0 (0.0)	1 (1.3)	0 (0.0)	0 (0.0)
Death	2 (2.4)	0 (0.0)	1 (1.1)^d^	0 (0.0)	0 (0.0)	0 (0.0)

^a^The total number of subjects who received at least 3 doses of study drug.

^b^None of the subjects who reported a level 4 AE had a level 3 AE.

^c^Includes 1 subject with an SAE of visual disturbance who discontinued after 1 dose (followed to the end of the study period by an ophthalmologist, but did not complete the study follow-up) and 1 subject with an SAE of erythema multiforme who discontinued after 2 doses (followed to the end of the study period and completed the study follow-up). A third subject experienced staphylococcal scalded skin syndrome after receiving 3 doses and also completed the study follow-up. All 3 subjects are included in the safety population.

^d^This subject withdrew consent on study day 13 and died on study day 153.

### Adverse Events Judged by the Site Investigators as Related to Study Treatment

Overall, the frequencies of AEs deemed by the investigator to be related to treatment with palivizumab or motavizumab were comparable among all 3 treatment groups (M/P, 16.9%; P/M, 19.3%; motavizumab only [control], 22.6%). The 2 most common AEs judged by the investigator to be related to study drug were injection-site erythema (M/P, 2.4%; P/M, 10.8%; motavizumab only, 8.6%) and irritability (4.8%, 7.2%, and 7.5%, respectively). The rate of injection-site erythema was significantly lower in the M/P group compared with the P/M group (*P *< 0.05), and the events were balanced before and after dose 3. These AEs were all level 1 in severity except in 2 subjects in the M/P group, each of whom experienced 1 level 2 event. All other drug-related AEs were level 1 or level 2 in severity, with the exception of 1 SAE of abnormal liver function tests in the motavizumab-only (control) group that was reported by the investigator at the day 150 study visit as level 4 in severity (reported as having returned to normal as of 142 days after the child received the fifth and final dose of motavizumab).

### SAEs Judged by the Site Investigators as Related to Study Treatment

Three SAEs were considered to be possibly or probably related to study treatment by the site investigators: visual disturbance, erythema multiforme (1 subject each; M/P group), and abnormal liver function tests (1 subject, motavizumab-only [control] group). Each subject had received only motavizumab before the onset of each SAE. The child with the reported event of visual disturbance was a 5-month-old male with a gestational age of 26 weeks, and a medical history of respiratory distress syndrome, bronchopulmonary dysplasia, necrotizing enterocolitis, gastroesophageal reflux, and intestinal obstruction. He had an initial retinopathy of prematurity screening at 6 weeks of age that was normal. He had received only 1 dose of motavizumab before the event. Two days after receiving motavizumab, the child was noted to have abnormal roving eye movements, which were random and not associated with any alteration in sensorium or vital signs. The event of visual disturbance was reported by the investigator as an SAE of level 2 severity and was judged to be possibly related to study treatment because of the temporal relationship, and study drug was permanently discontinued. The child was withdrawn from the study but was monitored by an ophthalmologist through study day 150, with the visual disturbance still present. There was no formal diagnosis of his visual abnormality. The child with the event of erythema multiforme was a 2-month-old male with a gestational age of 34 weeks and a history of wet lung, hyperbilirubinemia, bilateral pyelectasis, anemia of prematurity, and seborrheic dermatitis. He had previously taken cefadroxil, but not for the preceding 10 days. Two days after the second dose of motavizumab he experienced an abdominal rash. Four days after receiving motavizumab he presented to the investigational site with a generalized rash that was felt to be consistent with erythema multiforme. There were no respiratory symptoms or mucosal involvement, and his general condition was good. A diagnosis of erythema multiforme minor was made. He was treated with chlorpheniramine with resolution of the rash 2 days later. This event was reported as an SAE of level 2 severity and judged by the investigator to be probably related to study treatment, and study drug was permanently discontinued after its occurrence.

Study treatment was permanently discontinued because of an AE in a total of 3 patients (all in the M/P group). These included the events of visual disturbance and erythema multiforme, which are described above, and another child who experienced a level 3 SAE of staphylococcal scalded skin syndrome, which the investigator considered remotely related to study treatment. This event occurred 3 days after this child received the third dose of study drug (2 doses motavizumab and 1 dose palivizumab). The child's condition improved quickly and resolved after receiving antibiotics for bullous impetigo, and a dermatologist and infectious disease consultant felt that the clinical picture was consistent with staphylococcal scalded skin syndrome. This child and the child with erythema multiforme were monitored through the end of the study period.

### Deaths

There were 2 deaths during the study (one case of pneumonia and one case of sepsis). Both subjects were in the M/P treatment group and both deaths occurred before receiving palivizumab. The child who died from pneumonia was born prematurely at 29 weeks' gestation with a medical history significant for CLD, apnea of prematurity, and previous infection. She received 2 doses of motavizumab, the last dose administered 13 days before her death. She was found not breathing on the morning of her death; autopsy findings were consistent with acute bronchopneumonia. The child who died from sepsis had a gestational age of approximately 35 weeks with a history of suspected sepsis at birth. She had received 1 dose of motavizumab 3 days before her death at 10 days of age. On the day of her death, she was found not breathing while sleeping in bed with her parents; autopsy findings were consistent with sepsis and included hepatitis, septic adrenalitis, and neonatal pneumonia. Neither child had a known diagnosis of RSV at the time of death, and both deaths were considered by the investigator to be unrelated to study treatment. A third child died on study day 153 of an intestinal obstruction. The child had received 1 dose of motavizumab at the time of withdrawal. This death occurred outside of the study period, 140 days after withdrawal of consent and beyond the AE follow-up period (day 150). It was not considered by the site investigator to be related to study drug and is presented here for completeness.

### Antimotavizumab and Antipalivizumab Antibodies

**Figure 2:**
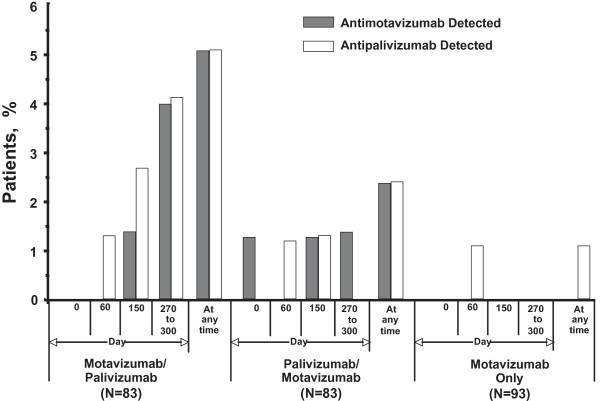
**Antimotavizumab and antipalivizumab antibodies detected during the study**. Antimotavizumab titers ranged from 1:40 to 1:1250 in the mixed motavizumab/palivizumab treatment group and from 1:10 to 1:250 in the mixed palivizumab/motavizumab treatment group. Final antibody assessments at days 270 to 300 correspond to 120-150 days after the final dose.

Few subjects developed detectable antibodies to either study drug; ADA titers observed at different time points during the study are illustrated in Figure 2. A total of 13 subjects (M/P, n = 8; P/M, n = 4; motavizumab only, n = 1) had detectable antipalivizumab and/or antimotavizumab antibody at any time during the study. All antimotavizumab antibodies were detected at study day 150 and/or 270-300 (except 1 subject in the P/M group who had antimotavizumab antibody detected at baseline prior to receiving study drug, and had no subsequent antibody detection after exposure to study drug). The subject in the M/P group with the SAE of erythema multiforme on study day 28 described above had antimotavizumab antibody of 1:50 detected on study day 150. Antimotavizumab antibody titers ranged from 1:40 to 1:1250 in the M/P treatment group and from 1:10 to 1:250 in the P/M treatment group. Antipalivizumab antibody titers were also low, ranging from 1:10 to 1:20 and were generally detected at study day 150 and/or 270-300. Cross-reactivity was low (1.1-1.3%). Only 1 subject each in the M/P and the motavizumab-only (control) treatment groups had detectable antipalivizumab antibodies without receiving palivizumab. The antipalivizumab titers of these subjects were low (≤1:20).

### Serum Drug Trough Concentrations

**Figure 3:**
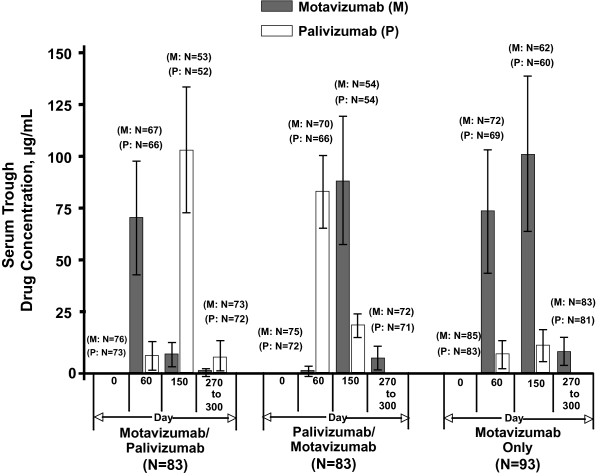
**Serum trough concentrations for motavizumab and palivizumab**. Data shown are mean (SD). The peak serum trough concentrations for each group reflect the sequence of dosing for that treatment group. Final serum concentrations (days 270-300) reflect those assessed 120-150 days after the final dose. Serum trough concentrations below the lower limit of quantification (motavizumab, 1.563 μg/mL; palivizumab, 10 μg/mL) were reported as 0.

Mean serum trough concentrations of motavizumab and palivizumab for each treatment group are illustrated in Figure 3. As expected, serum concentrations of each drug increased with treatment. On study day 60, mean serum trough concentrations of motavizumab in the M/P and motavizumab-only (control) groups were 74.74 μg/mL and 78.02 μg/mL, respectively. On study day 150, mean serum trough concentrations of motavizumab in the P/M and motavizumab-only (control) groups were 93.05 μg/mL and 105.8 μg/mL, respectively. For palivizumab, the mean serum trough concentration in the P/M group was 87.37 μg/mL on day 60.

## Discussion

In this small study evaluating the safety and tolerability of motavizumab and palivizumab administered sequentially during the same season for RSV prophylaxis, both study drugs were found to have an acceptable safety profile. Over 90% of subjects in the 3 treatment groups received all 5 doses of study drug and completed the study. Overall, the rates of AEs were similar among all 3 treatment groups. The most frequent AEs were infectious in nature, and were events common to and expected in this high-risk pediatric population. Of interest was the increased rate of level 3 AEs and SAEs in the M/P treatment group compared with the other treatment groups. This difference was present both before dose 3 (motavizumab for 2 doses) and after dose 3 (palivizumab for 3 doses), suggesting a cause other than receipt of both study drugs. In addition, before the switch from motavizumab to palivizumab, the rates of level 3 AEs as the highest severity, level 4 AEs, SAEs, SAEs judged by the site investigator to be related to study drug, and AEs that resulted in discontinuation of study drug were higher among children in the M/P group than in the motavizumab-only (control) group, again suggesting a difference that could not be explained by study treatment since both groups had received the same drug and number of doses.

When the increased incidence of SAEs in the M/P treatment group was further evaluated, it was noted that the increase in either time period was attributable to SAEs in the MedDRA "infections and infestations" system organ class. Since this was not an efficacy study, formal study data on RSV infections were not collected. However, some sites performed local RSV testing and reported the results. Of the 10 children in the M/P group with SAEs of respiratory infections who had local viral testing reported, 2 tests were positive for RSV. In addition, half of the SAEs in the M/P group occurred before dose 3 (ie, when subjects had received only motavizumab). For the 3 SAEs that were judged by the site investigators to be related to study treatment, all occurred after the receipt of motavizumab only.

Overall, antimotavizumab and antipalivizumab antibody detection was infrequent. A total of 13 subjects had antipalivizumab and/or antimotavizumab antibody at any time during the study, and no antimotavizumab antibody was detected in any subject in the motavizumab-only (control) treatment group. Mean serum trough concentrations of motavizumab and palivizumab in the mixed-dose and motavizumab-only (control) treatment groups were comparable based on the number of doses received. Only one child had a reported event consistent with a hypersensitivity reaction (erythema multiforme minor) after 2 doses of motavizumab. This event subsequently resolved; further study dosing was discontinued by the site investigator. ADA was detected in this child on study day 150, 124 days after final dose on study day 26. The causal relation of ADA with the event cannot be determined. No other events that could be considered immune-mediated were noted in children with and without ADA.

The conclusions drawn from this study are limited by the small sample size per group. Yet, in this group of high-risk children, the rates of AEs, serum drug trough concentrations, and occurrence of ADA associated with the sequential administration of motavizumab and palivizumab appear comparable to those observed in subjects administered motavizumab alone during the same RSV season.

While it did not address effects of sequential dosing, a large clinical study by Carbonell-Estrany et al recently compared the safety and efficacy of motavizumab (n = 3315) to palivizumab (n = 3298) and identified no significant overall difference in subjects reporting at least 1 AE (motavizumab, 85.6%; palivizumab, 86.0%), nor in subjects reporting at least 1 SAE (motavizumab, 14.6%; palivizumab, 15.3%) [20]. The overall rates of AEs and SAEs observed in the study by Carbonell-Estrany et al are similar to those in our study. Regarding AEs in the MedDRA system organ class of "infections and infestations," the study by Carbonell-Estrany et al found rates that were comparable for motavizumab and palivizumab. The children who received motavizumab had an overall AE infection rate of 66.3% with an SAE infection rate of 8.2%. In the palivizumab group, the overall AE infection rate was 68.3% with an SAE infection rate of 9.3% (G.A. Losonsky, MedImmune, LLC, personal communication). It is likely that differences among groups in our study could be attributed to the small sample size and not to the study drug administered or the sequence of administration.

## Conclusions

In this small study, motavizumab and palivizumab administered sequentially during the same RSV season exhibited an acceptable safety profile in high-risk preterm children. These results suggest that the safety, serum drug trough concentrations, and ADA profiles of palivizumab and motavizumab given in varying sequence are comparable to those observed in subjects receiving only motavizumab during the season.

## Competing interests

Dr. Fernández received a research grant from MedImmune.

Dr. Trenholme received research funding from MedImmune and Wyeth.

Dr. Abarca has received consultation/research grants from MedImmune.

Dr. Griffin, Dr. Losonsky, Ms. Hultquist, and Mr. Harris are employees of MedImmune.

## Authors' contributions

PF, AT, and KA were involved in the collection of data and interpretation of the results. MPG, MH, BH, and GAL were involved in the design of the study, analysis of the data, and interpretation of the results. All authors critically revised the manuscript and read and approved the final version.

## Pre-publication history

The pre-publication history for this paper can be accessed here:

http://www.biomedcentral.com/1471-2431/10/38/prepub
